# Current Status of the Management of Idiopathic Condylar Resorption/Progressive Condylar Resorption—A Scoping Review

**DOI:** 10.3390/jcm13133951

**Published:** 2024-07-05

**Authors:** Eiji Tanaka, Louis G. Mercuri

**Affiliations:** 1 Department of Orthodontics and Dentofacial Orthopedics, Tokushima University Graduate School of Biomedical Sciences, Tokushima 770-8504, Japan; 2 Department of Orthopaedic Surgery, Rush University Medical Center, Chicago, IL 60612, USA; louis_g_mercuri@rush.edu

**Keywords:** idiopathic condylar resorption, management, progressive condylar resorption, treatment

## Abstract

The temporomandibular joint (TMJ) is a complex synovial joint shrouded in mystery, as the etiology of many TMJ disorders are unsolved. Idiopathic/progressive condylar resorption (ICR/PCR) is one such TMJ disorder characterized by a gradually deteriorating mandibular condylar mass, resulting in severe mandibular retrognathia, which often accompanied by clockwise rotation of mandible and an anterior open bite. Since the etiology of the ICR/PCR remains unclear, no definitive prevention or management options have been established. To date, various symptomatic non-surgical, surgical, and salvage management options have been developed and reported. To understand the current status of the ICR/PCR management options, this article provides an overview of the options presently reported in the literature to reduce its TMJ symptoms and improve mandibular function and form.

## 1. Introduction

Idiopathic/progressive condylar resorption (ICR/PCR) is defined as a condition in which the mandibular condyle is specifically and progressively resorbed, accompanied by a marked reduction in mandibular ramus height. The reduction in the mandibular ramus height results in mandibular retrognathia and an anterior open bite, leading to an occlusal and masticatory/esthetic musculo/skeletal imbalance [1]. Since ICR/PCR is reported to occur most commonly in teenage females, growing ICR patients are likely to be diagnosed as simple maxillary protrusion or mandibular retrusion and receive inappropriate orthodontic management, which may induce the exacerbation of the mandibular condylar resorption [1,2,3].

The temporomandibular joint (TMJ) is functionally load bearing, and hence is subjected to a combination of compression, tension, and shear stresses [4,5]. Therefore, possible theories for the etiology of ICR/PCR include a combination of chemical and mechanical transduction processes [6]. The chemical and mechanical transduction might play a critical role in either reducing or exceeding the TMJ’s adaptive capacity threshold [7,8].

Although the specific cause of ICR/PCR has not been clearly identified, its strong predilection for teenage girls in their pubertal growth phase supports the theory of hormonal mediation. Excessive or deficient serum levels of β-estradiol are thought to be essential for ICR/PCR onset [9]. Elevated serum β-estradiol has been shown to have a synergistic or additive adverse effect on the articular cartilage including the articular disc [10,11,12]. Serum β-estradiol has an osteoprotective effect in enhancing osteoprotegerin expression and decreasing osteoclast activity. Therefore, reduced estrogen may also predispose patients to a bone degenerative process [9]. This means that estrogen exerts dual effects on mediating mandibular condylar fibrocartilage and subchondral bone turnover, with β-estradiol being predominant in females during the reproductive years [12]. Furthermore, estrogen receptors have been identified in the TMJs of female primates [13]. An increase in receptors may predispose patients to an exaggerated response to joint loading from parafunctional activity, trauma, orthodontics, or orthognathic surgery.

Once the breakdown of the joint starts, ICR/PCR can be crippling, leading to a severe dentofacial morphological deformity. Early diagnosis and management of the skeletal changes may help to avoid the outcome of deleterious skeletal change and an unsalvageable TMJ. Thus, as ICR/PCR exhibits mild to severe condylar resorption, management options can range from noninvasive therapy to minimally invasive, and invasive surgery according to its severity. Regardless of the severity, management goals include restored mandibular function, pain reduction, and improved dentofacial esthetics. However, there are no published randomized clinical trials that compare the outcomes of the various ICR/PCR management options [14]. To understand the current status of the ICR/PCR management options, this article provides an overview of the options presently reported in the literature to reduce the associated TMJ symptoms and improve mandibular function and form.

## 2. Methods

### 2.1. Search Strategy

The electronic databases PubMed, SCOPUS, and CiNill were searched to retrieve relevant articles published from January 1990 to March 2024 using the following terms “idiopathic OR progressive condylar resorption” AND “treatment OR management”. The last search was conducted in April 2024.

### 2.2. Study Selection

**Figure 1:**
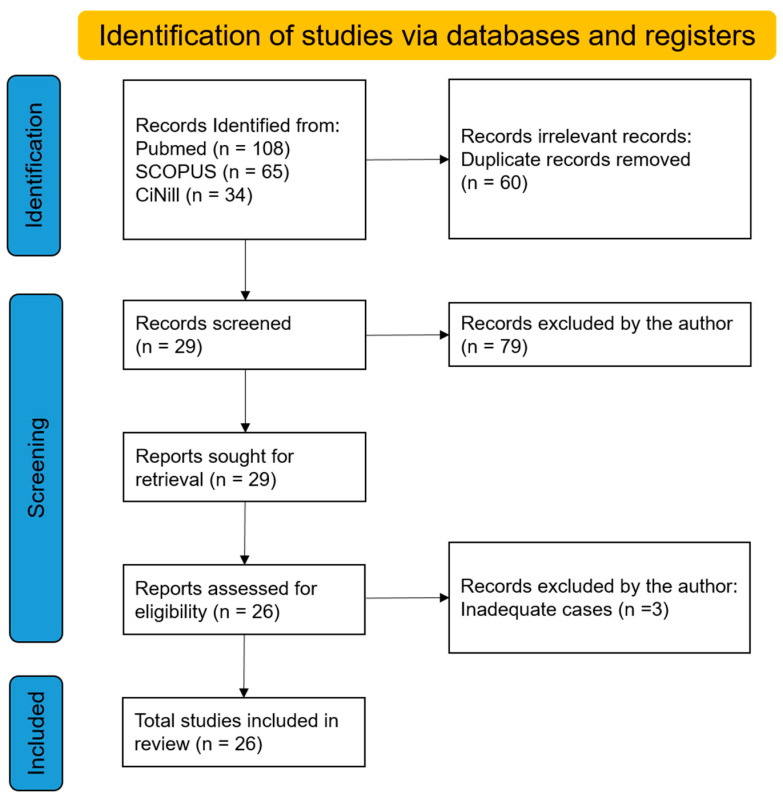
Preferred Reporting Items for Systematic Reviews and Meta-Analyses (PRISMA) flowchart.

One of the authors (ET) reviewed all of the abstracts. Articles responding to the following criteria were included and further analyzed: full text in English language; original article; human clinical trials; case report; and case series with a precise definition of the TMJ. Review articles, animal studies, opinion articles, and studies on syndrome- or systemic-disease related condylar resorption were excluded. The authors performed data retrieval and the quality and bias of the retrieved articles were not interpreted. Figure 1 shows the number of records identified and excluded from each database or registry searched.

## 3. Results

### 3.1. Search Results

From the titles and abstracts obtained from all of the databases we evaluated, the search strategy extracted a total of 108 articles. After reading the abstracts, seventy-nine articles were excluded, and the full-text articles of the remaining twenty-nine studies published in English were separately examined for eligibility by one author (ET), with three case reports being excluded: one article showed a case that received bimaxillary orthognathic surgery to reposition the prosthetic joints previously placed; the remaining two case reports suggested that it may not be idiopathic condylar resorption. Finally, 26 articles satisfied the criteria for inclusion and were processed for critical evaluation.

Total of 26 articles concerning 238 cases met the eligibility criteria. Managements included oral appliances, orthodontic management, orthognathic surgery with and without disc repositioning, mandibular osseodistraction, condylectomy with costochondral graft (CCG), and alloplastic total joint replacement (Table 1).

### 3.2. Confirmation of ICR/PCR Stability

Determination of whether the condylar resorption is active or inactive is critical for defining the appropriate timing and management option. Hatcher [6] recommended two imaging strategies for patients with PCR to determine stability. One was the use of nuclear medicine scanning, resulting in immediate results. The nuclear medicine approach commonly uses a bone scan imaging technique, such as technetium-99m methylene-diphosphonate (^99m^Tc-MDP) standard bone scans, and Tc-MDP single-photon emission CT (SPECT) [40,41]. Although bone scans are useful for evaluating some medical conditions, their specificity for condylar resorption may not be sufficient to determine stability [42]. The pathognomonic loss of the cortical layer of the condyle, which is typically detected in the erosion stage of ICR, can be evaluated by both CT and MRI [43]. Furthermore, CBCT imaging may be able to show the localization of the erosion and allow for the quantification of previously identified cases of ICR/PCR [43]. Both CT and CBCT are suitable for diagnosing the initial presentation and the progression of ICR [28,44]. Due to its higher soft tissue contrast resolution compared to CBCT, CT may be particularly suitable for patients in whom the exclusion of other differential diagnoses is especially important [45,46]. On the contrary, Hilgenberg-Sydney et al. indicated through a systematic review that CBCT could be a better imaging technique for the evaluation of degenerative joint diseases in the TMJ compared to CT [47]. Recently, Ibald et al. [48] attempted to establish reference values for mandibular dimensions in women using 158 MRI taken from women aged 15 to 40 years. However, they concluded that it is difficult to find new reference values for the mandibular morphology of ICR/PCR patients using an MRI.

Another strategy is to reevaluate and compare the condylar morphology after specific time periods. Time is the most useful tool in the determination of TMJ bony stability in PCR [6,31,49]. Once the radiographic features from the CT or comparable imaging of end-stage condylar resorption have been obtained, it appears to be prudent to wait 6–12 months to radiographically reevaluate stability in the TMJ [6]. However, follow-up for the remission stage may be too long and there are no guarantees that the resorptive process will not reactivate with the resumption of the management option chosen. Also, despite the presence of apparent risk factors or early signs of disease, onset may justify the adoption of a conservative and minimally invasive approach prior to surgical management, as there is no guarantee of definitive success in the early management of ICR/PCR despite this potentially promising approach.

### 3.3. Management of ICR/PCR

The management of ICR/PCR may be divided into non-surgical, invasive, or salvage modalities. The decision to surgically manage condylar resorption must be based on an evaluation of the patient’s response to non-surgical management and the patient’s concerns regarding their facial esthetics, their stomatological function, and the effect the condition has on the patients’ quality of life.

The non-surgical management modalities include oral appliances (one article with forty-two management cases), orthodontic management (two articles with twenty management cases), or the combination (two articles with fourteen management cases).

As for the invasive modalities, such as two-jaw orthognathic surgery with and without articular disc repositioning (one article with twenty-four management cases and five articles with forty-one management cases, respectively), and mandibular osseodistraction (one article with one management case) may be applicable.

The salvage modalities for end-stage ICR/PCR consist of condylectomy and reconstruction with autogenous tissue (five articles with sixty-four management cases), or alloplastic TMJ replacement (nine articles with fifty-two management cases).

### 3.4. Non-Surgical Management

Prior to invasive and salvage modalities, non-surgical options may be utilized, especially when ICR/PCR is in the active phase. Merkx and Van Damme [15] reported four patients with PCR who had previously undergone orthognathic surgery and then demonstrated PCR who were further managed with an oral appliance, had a functional occlusion, and tolerable TMJ complaints. Zhou et al. [36] evaluated condylar modelling in 42 patients with ICR treated with stabilizing oral appliances and demonstrated that the use of those appliances led to the tendency of the condylar surface for anabolic remodelling compared to ICR condyles without oral appliance therapy. In conclusion, they suggested the stabilization oral appliance therapy effectively reduced further bone destruction and promoted condylar remodelling.

However, oral appliance therapy is not critical for the management of ICR. According to Alsabban et al. [28], 81 out of 100 ICR patients had previously undergone one or more treatments that had failed. Among 81 patients with ICR, 19 patients (23%) had used an oral appliance, indicating that the use of such appliances may potentially worsen condylar resorption. It has been hypothesized that oral appliances can reduce the TMJ loading, and thereby halt condylar resorption progression and thereby prevent further bone degeneration [36,50,51].

Camouflage orthodontic management has been recognized as a non-surgical remedy for ICR/PCR. Hoppenreijs et al. [17] reported on the management of 13 PCR patients with non-surgical options including orthodontics and oral appliances in which satisfactory results were seen for all patients. To date, several cases treated with camouflage orthodontics have been published, in which counterclockwise rotation of the mandible using temporary anchorage devices (TADs) not only improved facial esthetics but also were suitable for condylar unloading [31,37,38]. Mao et al. [39] investigated TMJ stability and three-dimensional facial changes in 19 Class II hyperdivergent patients with inactive ICR after camouflage orthodontic management using TADs, and indicated that this management option for vertical control is acceptable for skeletal Class II hyperdivergent patients with ICR, thereby enabling improvement of the facial profile without surgery. Recently, Matsuki et al. [52] reported a severe ICR case treated orthodontically with TADs, and showed an excellent outcome with both functional and esthetic improvement. However, this case revealed an upward and backward displacement of the mandibular condyle after molar intrusion via TADs. The authors argued that this may be due to a noncongruent shape of the condyle-fossa relationship. It is well known that the human mandible functions as a third-order lever, and the mandibular condyle tends to function as the fulcrum of the lever [53,54]. The incongruency of the articular surfaces might prevent the mandibular condyle from acting as the fulcrum of mandibular rotation. Furthermore, Alsabban et al. [28] reported that 41 out of 81 ICR patients (52%) had undergone orthodontic management as the initial management for ICR that had failed.

Taken together, camouflage orthodontic management might be a promising remedy for inactive ICR followed by the use of an oral appliance [17,31]. Therefore, an oral appliance may provide adjunctive therapy for the management of ICR in combination with orthodontic and/or surgical management. However, further controlled studies are required.

### 3.5. Orthognathic Surgery

Many reports have been published in which revision surgery was performed for patients who had previously undergone orthognathic surgery and then developed PCR. However, the outcome of the remedial surgery was often reported to be unsatisfactory. Merkx and Van Damme [15] reported that the outcome of four PCR patients treated with revision surgery was unsatisfactory with poor esthetics and poor occlusal stability. According to Huang et al. [16], analyses of the 18 orthognathic surgery patients showed that relapse occurred in patients having bimaxillary surgery with mandibular advancements greater than 5 mm and with a preoperative posterior ramus height of less than 35 mm. Furthermore, orthognathic surgery in this small sample was associated with a complication rate (relapse or TMJ dysfunction) of approximately 45% (8 of 18). Al-Rezami et al. [55] demonstrated that significant condylar resorption following orthognathic surgery of the retrognathic mandible with a high mandibular plane angle is likely to occur regardless of the presurgical status of the condyle. Hoppenreijs et al. [17] managed thirteen PCR patients who previously underwent orthognathic surgery with second surgery, and showed that seven out of 1the thirteen 3 PCR patients exhibited good results, while the remaining six patients had a considerable relapse. Compared to the 13 PCR patients treated with non-surgical therapy, the success rate of second surgery was significantly lower.

Meanwhile, successful management outcomes of ICR/PCR patients with orthognathic surgery have been reported in which various innovations to the surgical technique could have led to a stable outcome [24,26,27]. Kau and Bejemir [26] performed two-jaw surgery including a segmental Le Fort I osteotomy and inverted L-shaped mandibular osteotomy with iliac bone grafting to a ICR patient, and resolved the functional, esthetic, and pain concerns of the patient to a satisfactory level. They noticed the importance of a three-dimensional simulation of the surgical process and orthodontic management for increased positive outcomes. Nakamura et al. [27] treated an ICR patient with maxillary osteotomy alone and concluded that severe mandibular retrognathia with ICR can be effectively treated without surgical mandibular advancement, thus decreasing the risk of postoperative PCR.

Wolford and his colleagues developed a Mitek bone anchor with osteointegration potential for articular disc stabilization [56,57,58]. Patients with active TMJ disease and either concomitant or resultant maxillofacial skeletal discrepancies, treated only with orthognathic surgery, often have poor outcomes and significant relapse [34,59,60,61]. This implies that patients with presurgical TMJ symptoms requiring mandibular advancement appear to be at an increased risk for condylar resorption. The most common TMJ pathology is anterior displacement of the articular disc, which can initiate a cascade of events leading to arthritis and other TMJ-related symptoms. Advancing the mandible in ICR/PCR patients with displaced discs will cause the discs to remain displaced as the condyles will seek the superior and posterior position in the fossa as a result of postsurgical soft tissue tension. Maxillomandibular advancement with counterclockwise rotation of the occlusal plane is a stable procedure for patients with healthy TMJs and for patients undergoing simultaneous TMJ disc repositioning using the Mitek anchor technique [60]. Galiano et al. [34] evaluated management outcomes for ICR patients treated with orthognathic surgery following articular disc repositioning with Mitek anchor technique, and reported that all 24 patients had good skeletal and occlusal stability as well as an improvement in TMJ pain, facial pain, and headaches with an improvement in their stomatognathic function.

In summary, it has been accepted that orthognathic surgery to manage mandibular retrognathia and maxillomandibular advancement with counterclockwise rotation of the occlusal plane is a stable procedure for mandibular retrognathia. However, patients with ICR/PCR treated with simple orthognathic surgery alone have poor outcomes and significant relapse, leading to more severe condylar resorption than they had preoperatively. Wang et al. [30] suggested that the mandibular advancement might be limited to 5 mm for patients with preoperative condylar resorption. This indicates that the choice of surgical procedure should be tailored to the preoperative TMJ conditions of each patient with ICR/PCR.

### 3.6. Total Joint Reconstruction

Condylectomy and reconstruction with either autogenous materials, for example, costochondral grafts (CCG), or alloplastic materials, represent other management modalities for ICR/PCR patients. Huang et al. [16] reported the results of condylectomy and reconstruction with a CCG in five ICR/PCR patients and concluded that condylectomy and CCG appeared to produce stable and functional results. Troulis et al. [18] reported endoscopic condylectomy and CCG reconstruction in seven ICR patients, and obtained satisfactory clinical outcomes with significant short-term morbidity. Furthermore, Troulis et al. [21] reported a retrospective study of 15 patients who had active, bilateral ICR which was surgically managed utilizing CCG condylar reconstruction and demonstrated that all patients showed stable and reproducible Class I occlusions without a significant relapse. Qui et al. [22] also reported 12 cases of ICR treated with condylectomy and CCG reconstruction, and suggested that patients with ICR had successful reconstruction of the condyles with endoscopic CCG. Recently, Peacock et al. [32] evaluated the long-term outcomes of the management for 25 patients with active ICR and demonstrated that stable and predictable long-term outcomes can be achieved using endoscopic condylectomy and CCG reconstruction.

CCGs are theoretically advantageous for reconstructing the TMJ for growing patients, but multiple shortcomings were reported, including the need for a secondary surgical site, with associated potential morbidity; chest wall deformity; scoliosis; and the risk of unpredictable growth [62,63,64,65]. Furthermore, CCGs do not allow for large mandibular movements, are unsuitable for increasing posterior mandibular vertical dimension, include remodelling and resorption that lead to occlusal changes, and do not provide the stability, esthetics, or quality of jaw function that a properly implanted, prosthetic, custom-made, patient-fitted alloplastic total joint reconstruction delivers. Therefore, it seems that an ICR surgical management option that does not depend on the compromised mechanical and biological adaptive capacity of the condyloid process of the mandible, or autogenous tissues (CCG) should be considered.

Management of ICR using alloplastic total TMJ reconstruction devices has been proposed. Alloplastic reconstruction is reported to be the most frequently preferred ICR/PCR surgical management option for failed prior non-surgical, orthognathic surgery, or autogenous bone grafts [28]. Alloplastic reconstruction allows for immediate postoperative rehabilitation without donor site morbidity. Several groups have reported satisfactory results using bilateral alloplastic reconstruction for the management of ICR/PCR. Mercuri firstly reported that five out of eight ICR patients received total alloplastic TMJ reconstruction, leading to long-term stable skeletal and occlusal results [19]. Mehra et al. [14] evaluated the clinical outcomes in patients after alloplastic TMJ replacement for the management of ICR using a retrospective analysis of 21 patients and showed that use of alloplastic joint prostheses allows for the execution of large mandibular advancements in a predictable and accurate manner with a significant decrease in TMJ symptoms. Further, several management cases with alloplastic joint reconstruction have been reported in which this salvage modality can provide a satisfying outcome [23,25,28,29,30,33]. Recently, Mercuri and Handelman [35] summarized the advantages and disadvantages of alloplastic total TMJ reconstruction considering the outcomes of 15 ICR/PCR cases treated with non-surgical or salvage modalities. Advantages include the ability to perform larger advancements with a custom prosthesis and the elimination of joint tissue that could have a role in disease progression, while disadvantages include the high cost of the device and surgery, potential mechanical wear and failure, uncertainty about long-term stability, and the difficulty of device application to skeletally immature patients [35,66].

## 4. Conclusions

Non-surgical, surgical, and salvage ICR/PCR management options have been developed, and their short- and long-term stability have been reported. However, many ICR/PCR patients have undergone one or more management options that have failed. This is because ICR/PCR management is still controversial and the development of clinical guidelines for ICR/PCR management have not yet been initiated. Priority should be given to identifying the causes of ICR/PCR and developing prophylactic or protective management options. Furthermore, early detection and diagnosis of ICR/PCR patients should garner greater attention from the basic scientific and clinical communities of interest, leading to the development of options for the prevention of the progression of the condylar resorption and its clinical sequelae. Until that happens, efforts should be made to alleviate symptoms and prevent the aggravation experienced by ICR/PCR patients by utilizing and reporting the outcomes of symptomatic therapies including camouflage orthodontic management with oral appliances, orthognathic surgery with and without disc repositioning, or total alloplastic joint reconstruction with autogenous or alloplastic materials in prospective controlled trials.

## Figures and Tables

**Table 1:** Studies describing the management and/or treatment of ICR/PCR.

Authors	Year	Country	Number	Management Method	Follow-Up	Major Results of Patients
Merkx et al. [15]	1994	The Netherlands	8	Orthognathic surgery	3.5 mo.–3 yr.	Revision surgery for treatment of ICR was unsatisfactory with poor esthetics and poor occlusal stability.
Huang et al. [16]	1997	USA	22	Condylectomy and CCG (5) or orthognathic surgery (18)	2 yr.	Condylectomy and CCG appeared to produce stable and functional results, during a short-term period, while orthognathic surgery might be associated with complication rate.
Hoppenreijs et al. [17]	1999	The Netherlands	26	Non-surgical therapies (13) or orthognathic surgery (13)	94 mo. for non-surgery; 58 mo. for surgery	13 patients treated with non-surgery showed satisfactory results, while 7 out of 13 patients treated with second surgery exhibited good results but the remaining 6 showed a considerable relapse.
Troulis et al. [18]	2004	USA	7	Endoscopic condylectomy and CCG	17 mo. (8–38 mo.)	Endoscopic condylectomy and CCG produce satisfactory clinical outcomes without significant morbidity in a short term period.
Mercuri [19]	2007	USA	8	Alloplastic TMJ reconstruction	Over 4 yr.	Total 5 of 8 ICR patients received TMJ reconstruction with a long term stability.
Schendel et al. [20]	2007	USA	1	Mandibular distraction osteogenesis	4 yr.	Mandibular osseodistraction produces stable results and no reactivation of ICR with a long-term stability.
Troulis et al. [21]	2008	USA	15	Condylectomy and CCG	Min. 12 mo.	A stable and satisfactory outcome is achievable in patients with active ICR treated by condylectomy and CCG reconstruction.
Qiu et al. [22]	2010	China	12	Reconstruction of the mandible with CCG	Min. 6 mo.	Patients with ICR had successful reconstruction of the condyle with endoscopic CCG.
Chung et al. [23]	2011	Republic of Korea	1	Alloplastic TMJ reconstruction	1 yr.	Total alloplastic joint reconstruction and counterclockwise rotation of maxillomandibular complex provided a satisfying outcome.
You et al. [24]	2011	Republic of Korea	1	Orthognathic surgery (Two-jaw surgery)	3 yr.	The case showed functional remodelling of the condyle in preoperative ICR.
Alexander [25]	2012	USA	1	Alloplastic joint reconstruction	16 yr.	The case treated with a single-stage surgery with alloplast with a 16-year follow-up.
Kau and Bejemir [26]	2015	USA	1	Orthognathic surgery (iliac bone graft with inverted L-osteotomy)	1 yr.	A segmental Le-Fort I osteotomy and inverted L-osteotomy with iliac bone grafting led to resolve functional, esthetic, and pain concerns to a satisfactory level.
Mehra et al. [14]	2016	USA	21	Alloplastic joint reconstruction	6.2 yr. (5–12 yr.)	Use of alloplastic joint prostheses allows for the execution of large mandibular advancements in a predictable and accurate manner with a meaningful decrease in symptoms of TMJ dysfunction.
Nakamura et al. [27]	2016	Japan	1	Maxillary osteotomy alone	30 mo.	Mild ICR patient can be effectively treated without surgical mandibular advancement.
Alsabban et al. [28]	2018	USA	1	Alloplastic joint reconstruction	17 mo.	As a single surgery, treatment with total joint reconstruction showed stable result.
Rahman et al. [29]	2019	USA	1	Alloplastic joint reconstruction	0 mo.	A patient with bilateral ICR was treated with total joint replacement and orthodontic-orthognathic surgery.
Wang et al. [30]	2019	USA	1	Alloplastic joint reconstruction	0 mo.	A patient with bilateral ICR was treated with total joint replacement and orthodontic-orthognathic surgery.
Lee et al. [31]	2019	Republic of Korea	1	Orthodontic treatment after stabilization splint	2 yr.	After stabilization splint therapy, orthodontic treatment with TADs achieved an acceptable occlusion and facial esthetics.
Peacock et al. [32]	2019	USA	25	Endoscopic condylectomy and CCG	At least 3 yr.	Stable and predictable long-term outcomes can be achieved using endoscopic condylectomy and CCG for treatment of active ICR.
Chamberland [33]	2019	Canada	3	Alloplastic joint reconstruction	0.5–1 mo.	Two ICR patients after orthodontic treatment was managed by total joint replacement.
Galiano et al. [34]	2019	Brazil	24	Orthognathic surgery with disc repositioning	30.3 mo.	Adolescent condylar resorption can be successfully treated with disc repositioning and orthognathic surgery.
Mercuri and Handelman [35]	2020	USA	15	Total joint replacement	6 yr.	Among 15 patients with ICR/PCR, 12 patients underwent alloplastic TMJ replacement, and 11 out of 12 patients showed stable result. Among the remaining three, two patients refused treatment, and one showed PCR after occlusal appliance therapy.
Zhou et al. [36]	2021	China	42	Splint therapy	Min. 6 mo.	The anabolic modelling tendency of the condylar surface p was greater in the stabilization splint group than in the control group. The stabilization splint therapy effectively reduced further bone destruction and promoted condylar modeling.
Wang et al. [37]	2021	China	1	Orthognathic surgery (BSRO and genioplasty)	1 yr.	Facial appearance and occlusion improved significantly, and a stable result was obtained with a 1-year follow-up.
Noh and Park [38]	2021	Republic of Korea	1	Orthodontic treatment with TADs	2 yr.	Counterclockwise mechanics not only improved facial esthetics but were also suitable for condylar unloading.
Mao et al. [39]	2022	China	19	Orthodontic treatment with TADs	0	Orthodontic treatment with TADs resulted 2.27° counterclockwise mandibular rotation.
